# Acute and Long-Term Effects of Stretching with Whole-Body Vibration on Young’s Modulus of the Soleus Muscle Measured Using Shear Wave Elastography

**DOI:** 10.3390/sports12060165

**Published:** 2024-06-14

**Authors:** Hayato Miyasaka, Bungo Ebihara, Takashi Fukaya, Hirotaka Mutsuzaki

**Affiliations:** 1 Department of Rehabilitation, Tsuchiura Kyodo General Hospital, 4-1-1 Otsuno, Tsuchiura 300-0028, Ibaraki, Japan; bun.hirakata@gmail.com; 2 Graduate School of Health Sciences, Ibaraki Prefectural University of Health Sciences, 4669-2 Ami, Ami 300-0394, Ibaraki, Japan; 3 Department of Physical Therapy, Faculty of Health Sciences, Tsukuba International University, 6-8-33 Manabe, Tsuchiura 300-0051, Ibaraki, Japan; t-fukaya@tius.ac.jp; 4 Center for Medical Science, Ibaraki Prefectural University of Health Sciences, 4669-2 Ami, Ami 300-0394, Ibaraki, Japan; mutsuzaki@ipu.ac.jp; 5 Department of Orthopedic Surgery, Ibaraki Prefectural University of Health Sciences Hospital, 4773 Ami, Ami 300-0331, Ibaraki, Japan

**Keywords:** stretching, whole-body vibration, elastography, Young’s modulus, soleus muscle, stiffness, range of motion

## Abstract

The effect of whole-body vibration (WBV) stretching on soleus (SOL) muscle stiffness remains unclear. Therefore, we aimed to investigate the acute and long-term effects of stretching with WBV on SOL muscle stiffness. This study employed a repeated-measures experimental design evaluating 20 healthy young males. SOL muscle stretching with WBV was performed for 5 min per day (1 min per set, five sets) over 4 weeks, for 4 days a week. Participants stretched the SOL muscle with ankle dorsiflexion in a loaded flexed knee position on a WBV device. Data were obtained to examine acute effects before stretching, immediately after stretching, and at 5, 10, 15, and 20 min. Moreover, data were obtained to examine the long-term effects before stretching, immediately after the completion of the 4-week stretching program, and at 2 and 4 weeks later. SOL muscle stiffness was measured using Young’s modulus with shear wave elastography. The acute effect of SOL muscle stretching with WBV persisted for up to 20 min. Additionally, the long-term effect of stretching was better maintained than the acute effect, which was effective for up to 4 weeks (*p* < 0.001). Clinically, continuous stretching with WBV may be used to improve SOL muscle stiffness in rehabilitation programs.

## 1. Introduction

The soleus (SOL) muscle is the largest triceps surae muscle and a monoarticular plantar flexor muscle [1,2]. The SOL muscle originates from the upper half of the gastrocnemius and, together with the gastrocnemius muscle, forms the Achilles tendon (AT), terminating at the calcaneal tuberosity [3,4]. SOL muscle stiffness plays a role in the development of medial tibial stress syndrome [5]. The AT insertion from the SOL muscle is the most stretched during foot pronation and supination, in addition to dorsiflexion, potentially contributing to AT disorders [6]. Therefore, SOL muscle flexibility is important for preventing and improving sports-related injuries.

Whole-body vibration (WBV) is a contemporary fitness technology that is supplied using a suitable frequency and amplitude on a particular platform [7]. WBV causes rapid changes in muscle length due to mechanical vibrations transmitted when the subject is in contact with the WBV platform [8]. WBV is an intervention that may have beneficial effects on muscle flexibility and range of motion (ROM) [9]. Many processes, including the lowering of static and phasic stretch reflexes, raise the pain threshold, and causing the stretched muscle to relax may also contribute to the benefits of WBV [10]. Additionally, vibrations increase the intramuscular temperature and encourage an improved blood circulation [11]. Stretching with WBV is effective in improving the flexibility of hamstrings in healthy participants [9]. Moreover, stretching with WBV on the hamstrings of professional soccer players maintains longer-term effects [12]. Stretching with WBV is also effective in enhancing ankle ROM in patients with chronic ankle instability [13]. However, stretching programs have not been established, and the changes in SOL muscle stiffness before and after stretching with WBV remain unclear. Additionally, the duration of the acute and long-term effects of stretching with WBV remains unclear. It is necessary to objectively evaluate the effect of stretching with WBV to establish a stretching program that improves the flexibility of the SOL muscle.

Shear wave elastography (SWE) has been used to assess the stiffness of individual muscles [14], which was quantified using Young’s modulus. Young’s modulus measurements of the SOL muscle have exhibited high reliability [15]. Furthermore, SWE can be used before and after muscle stretching to evaluate the effectiveness of Young’s modulus [16]. Therefore, measuring Young’s modulus before and after stretching with WBV using SWE could be used to evaluate acute and long-term effects. The effects of stretching on the gastrocnemius muscle have been reported using SWE [17,18]. However, few studies have investigated the effects of stretching on the SOL muscle [19].

The first experiment aimed to quantitatively clarify the acute effects of stretching with WBV on SOL muscle stiffness. The second experiment aimed to observe the long-term effects of stretching with WBV on SOL muscle stiffness. We hypothesised that Young’s modulus would rapidly decrease after stretching and gradually increase toward the baseline in the acute phase. Furthermore, we hypothesised that Young’s modulus would decrease after stretching and be maintained in the long-term phase. Clarification of these effects may aid in planning stretching programs and optimising exercise therapies.

## 2. Materials and Methods

### 2.1. Participants

This study, conducted in 2024, recruited healthy, male hospital employees through hospital networks and posters. Twenty healthy young males participated in the study. These participants were not athletes. Regarding the physical activity level, participants are classified as Tiers 0 or 1 [20]. The right foot was measured in all participants. Previous studies have reported a high reliability in measuring the stiffness of the SOL muscle in the right foot [15]. The participants had an ankle dorsiflexion ROM of at least 10° and no joint or muscle pain. Participants with a history of neuromuscular disease or musculoskeletal injury in the lower extremities and those who completed less than 80% of the WBV stretching program were excluded.

This study was approved by the institutional ethics committee and was conducted in compliance with the Declaration of Helsinki.

### 2.2. SOL Muscle Stretching with WBV Protocol

**Figure 1:**
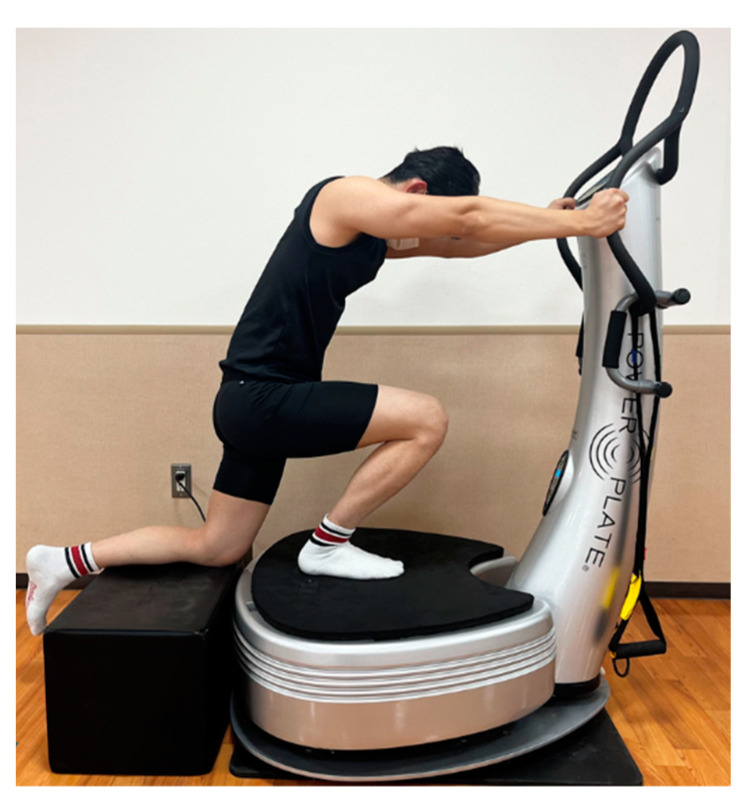
Illustration of the soleus (SOL) muscle stretching with the whole-body vibration (WBV) device. The participants stretched the SOL muscle during ankle dorsiflexion in the knee-flexed position under a load.

The participants stretched on a WBV device (Power Plate; Protea Japan K.K., Tokyo, Japan) [21]. The system vibrates along three axes (the X-, Y-, and Z-axes) and is set to a high-amplitude mode (2 mm) at a frequency of 30 Hz [22,23]. The participant placed the right leg on the WBV device and stretched the SOL muscle during ankle dorsiflexion in the knee-flexed position under a load (Figure 1). The participant placed the left foot on a block at the same height as the WBV device and held the device handle with both hands to maintain balance. WBV stretching was performed for 5 min/day (1 min/set, five sets) with a 5 s rest between each set. The participants performed SOL muscle stretching with WBV 4 days a week for 4 weeks (Figure 1). Participants stretched using WBV while the tester observed. Stretching was applied at a strength immediately before pain onset [24]. The participants underwent stretching barefoot, as wearing shoes also reduced neuromuscular responses to vibration [25]. Participants were instructed to avoid impact activities and sports 2 days before measurements and to maintain their normal activity level.

### 2.3. Measurement of Young’s Modulus of SOL Muscle and AT

Young’s moduli were measured using a 2–10 MHz linear transducer (Supersonic Imaging, Aix-en-Provence, France). The same physical therapist with 8 years of experience in musculoskeletal ultrasound tests evaluated Young’s modulus using the SWE opt penetration mode. The formula for Young’s modulus, E, is as follows:

E = 3ρc^2^; Young’s modulus, E; tissue density, ρ; shear wave velocity, c [26].

SOL muscle and AT have Young’s moduli of approximately 0–600 kPa and 0–800 kPa, respectively. The scanning was set at 2.5 cm depth and 2.0–3.0 cm focus or 1.0 cm depth and 0.5–1.0 cm focus in the SOL muscle and AT, respectively.

**Figure 2:**
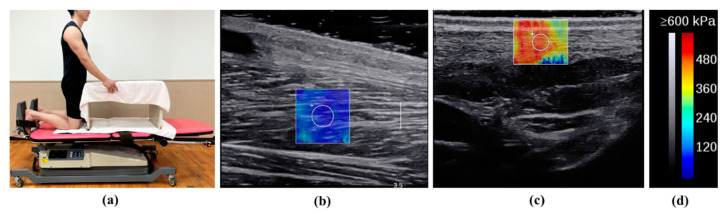
Illustration of the Young’s modulus measurement. (**a**) Posture during the measurement; (**b**) SWE images of the SOL muscle; (**c**) SWE images of the AT; (**d**) the colour scale.

Room temperature was maintained at 25℃ [27]. Participants were measured while kneeling, with knees flexed at 90°, the upper body supported by a table, and ankle dorsiflexion at 10° (Figure 2) [28]. The participants were instructed to relax during the measurements. Ultrasound images were captured along the longitudinal axes of the muscles and tendons. The measurement location of the SOL muscle was near the muscle–tendon transition of the gastrocnemius, and that of the AT was 3 cm above the calcaneal tuberosity [15]. These levels are considered clinically important because the stiffness of the SOL muscle and AT makes them prone to overuse damage [29,30]. Furthermore, their relatively superficial location allows Young’s modulus to be measured. B-mode horizontal-axis images were used to identify the musculotendinous transition zone, which was marked on the skin using a black pen. Region of interest (ROI) circles of 4 mm and 3 mm in diameter were used for the SOL muscle and AT, respectively, and were set near the centre of the SOL muscle and AT (Figure 2). A large amount of gel was used to reduce the effect of pressure on the skin. Young’s modulus measurements of SOL muscle and AT exhibit a high reliability [15].

**Figure 3:**
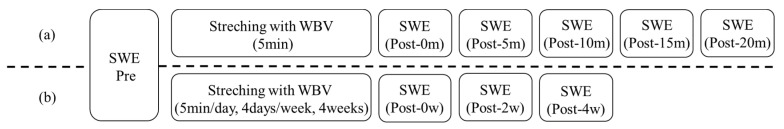
Experimental protocols 1 (**a**) and 2 (**b**). Pre, before stretching with WBV; Post-0m, immediately after stretching with WBV; Post-5m, after 5 min of stretching with WBV; Post-10m, after 10 min of stretching with WBV; Post-15m, after 15 min of stretching with WBV; Post-20m, after 20 min of stretching with WBV; Post-0w, immediately after the completion of the 4-week stretching program; Post-2w, 2 weeks after the completion of the 4-week stretching program; Post-4w: 4 weeks after the completion of the 4-week stretching program.

First, Young’s modulus (pre) was measured before WBV stretching. In experiment 1, as shown in Figure 3a, data were acquired at six time points (before stretching; immediately after stretching; and at 5, 10, 15, and 20 min [pre, post-0m, post-5m, post-10m, post-15m, and post-20m, respectively]) for each participant to examine the acute effects. The participants sat and relaxed between measurements. In experiment 2, as shown in Figure 3b, data were acquired at four time points (before stretching, immediately after the completion of the 4-week stretching program, and at 2 and 4 weeks later [pre, post-0w, post-2w, and post-4w, respectively]).

### 2.4. Measurement of Ankle Dorsiflexion ROM

Ankle dorsiflexion ROM was measured with the patient in the supine position and the knee in a 90° flexed position using a goniometer with a minimum value of 1°. During the measurement, the fulcrum of the goniometer was positioned at the centre of the lateral malleolus, the stationary arm was aligned with the long axis of the fibula, and the movement arm was parallel to the plantar surface of the foot.

### 2.5. Statistical Analysis

G*power 3.1 (Heinrich Hein University, Düsseldorf, Germany) was used to calculate the sample size required for multiple comparisons following one-way repeated analysis of variance [effect size = 0.25, α error = 0.05, Power = 0.80] [31], and the result was 19 [32]. Therefore, 20 healthy men were included in this study. All data were assessed for distribution using the Shapiro–Wilk test. Means and standard deviations were computed for normally distributed data, and medians and interquartile ranges were calculated for non-normally distributed data. In experiments 1 and 2, a one-way repeated analysis of variance was performed to evaluate changes in Young’s modulus of SOL muscle and AT over time, both before and after WBV stretching. Effect sizes (r) were calculated for Pre and all pairwise comparisons. Effect sizes, r, of 0.1, 0.3, and 0.5 were estimated as small, moderate, and large, respectively [31].

Moreover, the Bonferroni post hoc test was used to demonstrate the time-course effect. Statistical significance was set at *p* < 0.05. All statistical analyses were performed using SPSS^®^ Statistics version 29.0.2 (IBM Corp., Armonk, NY, USA).

## 3. Results

### 3.1. Participants’ Characteristics

Table 1 summarises the participants’ characteristics. The mean age of the participants was 27.1 ± 2.5 years. The completion rate of the WBV stretching program was 100% for all participants.

### 3.2. Changes in Young’s Moduli of the SOL Muscle and AT

**Figure 4:**
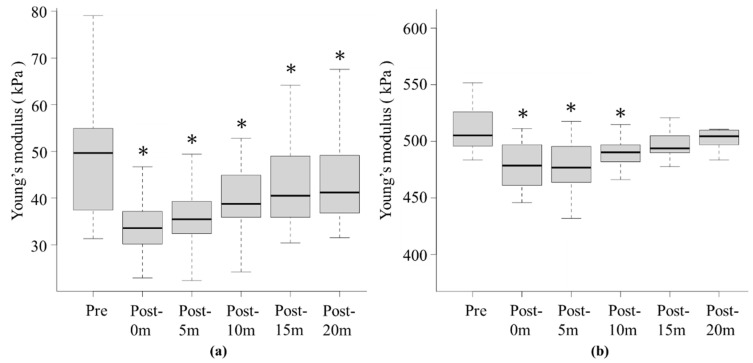
Change over time in Young’s modulus of SOL muscle (**a**) and AT (**b**) before and after stretching with WBV in experiment 1. * Significant differences were detected using one-way repeated measures analysis of variance and Bonferroni post hoc test after stretching compared with before stretching (*p* < 0.05). Black lines within boxes and box borders indicate the median, 25th percentile, and 75th percentile, respectively.

**Figure 5:**
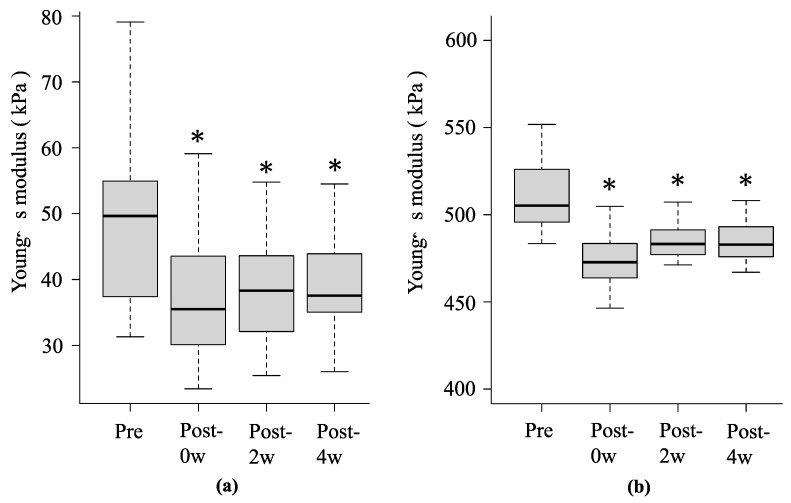
Change over time in Young’s modulus of SOL muscle (**a**) and AT (**b**) before and after stretching with WBV in experiment 2. * Significant differences were detected using one-way repeated measures analysis of variance and Bonferroni post hoc test compared to before stretching (*p* < 0.05). Black lines within boxes and box borders indicate the median, 25th percentile, and 75th percentile.

Figure 4 and Figure 5 summarise the changes in Young’s moduli of the SOL muscle and AT before and after stretching with WBV. In experiment 1, the mean Young’s modulus of the SOL muscle at pre, post-0m, post-5m, post-10m, post-15m, and post-20m were 49.0 ± 12.3 kPa, 34.7 ± 6.9 kPa (*p* < 0.001, r = 0.87), 35.5 ± 6.7 kPa (*p* < 0.001, r = 0.85), 39.7 ± 7.7 kPa (*p* < 0.001, r = 0.72), 41.9 ± 8.9 kPa (*p* < 0.001, r = 0.68), and 43.1 ± 9.2 kPa (*p* = 0.002, r = 0.65), respectively, and AT were 514.4 ± 29.8 kPa, 474.6 ± 30.0 kPa (*p* < 0.001, r = 0.88), 482.1 ± 32.1 kPa (*p* < 0.001, r = 0.87), 490.1 ± 15.1 kPa (*p* = 0.007, r = 0.71), 502.1 ± 23.8 kPa (*p* = 0.06, r = 0.60), and 512.9 ± 30.5 kPa (*p* = 0.23, r = 0.34), respectively (Figure 4a,b).

In experiment 2, the mean Young’s modulus of the SOL muscle at pre, post-0w, post-2w, and post-4w were 49.0 ± 12.3 kPa, 36.8 ± 9.2 kPa (*p* < 0.001, r = 0.87), 38.2 ± 8.7 kPa (*p* < 0.001, r = 0.87), and 38.9 ± 8.0 kPa (*p* < 0.001, r = 0.87), respectively, and AT were 514.4 ± 29.8 kPa, 474.3 ± 14.5 kPa (*p* < 0.001, r = 0.88), 484.4 ± 9.4 kPa (*p* < 0.001, r = 0.88), and 485.3 ± 12.4 kPa (*p* < 0.001, r = 0.88), respectively (Figure 5a,b).

### 3.3. Changes in Ankle Dorsiflexion ROM

**Figure 6:**
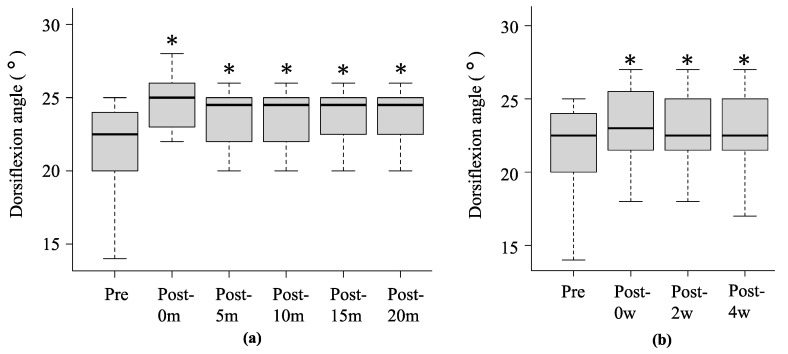
Change over time in ankle dorsiflexion ROM before and after stretching with WBV in experiment 1 (**a**) and experiment 2 (**b**). * Significant differences were detected using one-way repeated measures analysis of variance and Bonferroni post hoc test compared to before stretching (*p* < 0.05). Black lines within boxes and box borders indicate the median, 25th percentile, and 75th percentile.

Figure 6 summarises the changes in ankle dorsiflexion ROM in experiments 1 and 2. In experiment 1, the mean ankle dorsiflexion ROM at pre, post-0m, post-5m, post-10m, post-15m, and post-20m was 22.1 ± 3.6°, 23.3 ± 3.1° (*p* < 0.001, r = 0.73), 23.2 ± 3.1° (*p* < 0.001, r = 0.68), 23.1 ± 3.3° (*p* < 0.001, r = 0.68), 22.9 ± 3.3° (*p* = 0.001, r = 0.65), and 22.6 ± 3.4° (*p* = 0.001, r = 0.56), respectively. In experiment 2, the mean ankle dorsiflexion ROM at pre, post-0w, post-2w, and post-4w was 22.1 ± 3.6°, 24.8 ± 3.5° (*p* < 0.001, r = 0.86), 23.8 ± 3.4° (*p* < 0.001, r = 0.84), and 23.8 ± 3.3° (*p* < 0.001, r = 0.85), respectively.

## 4. Discussion

In this study, we aimed to determine the acute and long-term effects of stretching with WBV on Young’s modulus of the SOL muscle using SWE. The findings of this study support our hypothesis that continuous stretching with WBV effectively improves SOL muscle stiffness and ankle dorsiflexion ROM. We have shown that the acute effects of 5 min of SOL muscle stretching with WBV persisted for up to 20 min. Additionally, short-term stretching gradually increased SOL muscle stiffness, whereas continued stretching for over 4 weeks maintained the improvement in stiffness for up to 4 weeks.

SOL muscle stretching increases ankle dorsiflexion ROM [33]. Stretching has been reported to alter the perception of muscle tension and stiffness [34,35]. WBV also increases blood flow and intramuscular temperature [36]. Park et al. [37] demonstrated that static stretching with vibration reduced pain perception. This increase in pain threshold reduces pain and allows for further stretching. Previous studies on WBV suggest that it has been used as an effective way to improve flexibility in several sports [38,39,40], students [9,41], adults [42], and older people [43]. In addition, an increased flexibility in divers [44], dancers [45], and synchronised swimmers [46] has also been reported. In these reports, field tests such as sit-and-reach tests and ROM are often used to assess flexibility. Feland et al. [9] found that static stretching with WBV greatly improved flexibility. However, flexibility studies have focused primarily on the hamstrings in healthy populations. Few studies have measured the effects of stretching with WBV on Young’s modulus of the SOL muscle using SWE. In this study, SOL muscle stiffness was measured more directly using SWE.

The acute effect of 5 min static stretching on the plantar flexor tendon of the ankle joint has been reported to last 5–15 min [47,48]. The biomechanical effects of viscoelastic changes are also short-lived [34]. Additionally, short-term stretching is only related to changes in muscle structure, with no effect on tendon structure [48]. However, short-term stretching with WBV had a lasting effect on the SOL muscle for up to 20 min and an immediate effect on the AT. Vibration is thought to enhance the stretch reflex loop from a physiological point of view by activating the major terminals of the muscle spindles, which influences the contraction of the primary active muscle and concurrently inhibits the contraction of the antagonist muscle [49]. Stretching with WBV activated Ia inhibitory interneurons in the antagonist muscle, which may have reduced muscle tone and improved stiffness. Other studies have reported a positive correlation between static stretching of the gastrocnemius and SOL muscles and increased dorsiflexion ROM [50,51]. McKeon et al. [52] demonstrated a 1.15 ± 0° improvement in dorsiflexion ROM after one stretching session and a 1.24 ± 0° improvement after six 5 min stretching sessions over 2 weeks.

Long-term stretching with WBV is more effective than short-term stretching with WBV. These results suggest that repeated long-term stretching with WBV may affect muscle stiffness and ROM. Notably, long-term repetitive stretching has a cumulative effect [9,13,53]. Feland et al. [9] reported that WBV may enhance flexibility retention and is suitable as an adjunct to static stretching. Additionally, improvements in the Young’s moduli of the SOL muscle and AT were maintained. The AT attaches to the calcaneus as the stop tendon of the three-headed triceps surae, which is composed of the gastrocnemius and SOL muscles [54]. Stretching with WBV may have improved the stiffness of the SOL muscle, which also reduced the passive tension applied to the AT and improved Young’s modulus of the AT. The 95% confidence interval of the minimal detectable change of Young’s modulus of the SOL muscle and AT 10° ankle dorsiflexion was 10.1 kPa and 17.8 kPa, respectively [15]. In this study, the changes in Young’s moduli of the SOL muscle and AT after the 4-week stretching program were 12.2 kPa and 40.1 kPa, respectively. This result may reflect true differences that exceed measurement error.

Clinically, stretching with WBV may improve SOL muscle stiffness. Additionally, long-term stretching with WBV may be an effective intervention for rehabilitation and sports practice. The program in this study was found to remain effective for up to 4 weeks. Therefore, implementing this program once every 4 weeks may help maintain improvement in SOL muscle stiffness. However, prolonged stretching may cause a temporary reduction in muscle strength and performance [55]. Therefore, when incorporating stretching into the routine warm-up immediately before exercise, it should be of a short duration, or the time of day when the stretching is performed should be considered.

This study had some limitations. First, muscle activity may have affected Young’s modulus because SOL muscle activity was not monitored during measurement. Second, the study only included young, healthy males and the number of participants was small. Therefore, it remains unclear whether this finding applies to women, other age groups, and patients with sports injuries. Future research is warranted on those with injuries or AT disorders. Third, the control group was not included. These validations are necessary because few studies have compared the effects of stretching alone to stretching with WBV. Finally, the study did not measure the long-term effects beyond 4 weeks. Therefore, further research is required.

## 5. Conclusions

Young’s modulus of the SOL muscle decreased immediately after short-term stretching with WBV and was maintained for up to 20 min. Long-term stretching maintained SOL muscle stretching more effectively than short-term stretching, persisting for up to 4 weeks. Clinically, long-term stretching with WBV may be an effective rehabilitation program for improving SOL muscle stiffness.

## Figures and Tables

**Table 1:** Participants’ physical characteristics.

Age (years)	27.1 ± 2.5 ^a^
Height (m)	1.73 (1.70–1.76) ^b^
Weight (kg)	64.5 ± 6.3 ^a^
Body mass index (kg/m^2^)	20.7 (20.1–22.7) ^b^
Dominant leg (right/left)	19/1 ^c^

^a^ Values are presented as mean ± standard deviation. ^b^ Values are presented as median (interquartile range). ^c^ n/n.

## Data Availability

The data supporting the findings of this study are available upon request from the corresponding author. The data are not publicly available owing to restrictions on their containing information that could compromise the privacy of the research participants.
